# Durable strong efficacy and favorable long-term renal safety of the anatomically optimized distal renal denervation according to the 3 year follow-up extension of the double-blind randomized controlled trial

**DOI:** 10.1016/j.heliyon.2022.e08747

**Published:** 2022-01-13

**Authors:** Stanislav Pekarskiy, Andrei Baev, Alla Falkovskaya, Valeria Lichikaki, Ekaterina Sitkova, Irina Zubanova, Musheg Manukyan, Mikhail Tarasov, Viktor Mordovin, Sergei Popov

**Affiliations:** Cardiology Research Institute, Tomsk National Research Medical Center, Russian Academy of Sciences. 111a Kievskaya Street, Tomsk, 634012, Russia

**Keywords:** Arterial catheterization, Peripheral, Renal artery, Radiofrequency ablation, Essential hypertension, Blood pressure monitoring, Ambulatory

## Abstract

**Background:**

Historical reports on surgical renal denervation consistently describe renal plexus as a triangle or fan-like structure converging at the kidney gate. Following that anatomy, we developed a distal mode of radiofrequency renal denervation (RDN) mainly in segmental branches of the renal artery and confirmed its superior efficacy over the conventional main trunk procedure in a 6-months double-blind randomized controlled trial (NCT02667912). To assess the long-term effects of distal RDN we extended the follow-up of our study to three years.

**Methods:**

BP, serum creatinine, eGFR were measured one and three years after randomization; major adverse renal events were assessed over the entire study period. The blinding was maintained over the entire three-year study period.

**Findings:**

Of 55 randomized patients, 47 (23/24, distal/main trunk RDN, respectively) were assessed at one year and 39 (21/18) at three years post-procedure. Twenty-four-hour ambulatory systolic BP remained powerfully lowered after distal RDN both at one- and three-years assessments(mean change from baseline: -18.0, 95% CI -27.6 to -8.5; p<0.05 and -16·9, 95% CI -27·3 to -6·5; p<0·05, mmHg, respectively. This was accompanied by a moderate drop in eGFR at one year: -8·9 ml/min/m2, 95% CI -14·8 to -3·1; p<0·05, which, however, subsequently decreased in size at three years: -6·5, 95% CI -13·2 to 0·3; p>0·05. After main trunk RDN, the decrease of 24h systolic BP was quite moderate at one year: -12·1, 95% CI -19·2 to -5·0; p<0·05, and further weakened at three-year assessment: -8·5, 95% CI -19·7 to 2·2; p>0.05. eGFR was almost unchanged at one year: -1·3, 95% CI -6·6 to 4·0; p>0·05, but significantly decreased at three years: -5·0, 95% CI -9·6 to -0·3; p<0·05.

**Interpretation:**

Our data demonstrate the durable strong BP-lowering efficacy and favorable long-term renal safety of distal RDN.

## 1 Introduction

Transcatheter therapies are a completely new treatment paradigm. Instead of reaching the disease sites through traumatic surgical incisions or nonselective dissolution of drugs in the human body, transcatheter interventions deliver treatments through a lumen of the circulatory system, a framework of elastic tubes penetrating every part of the human body. The treatment is delivered selectively without trauma to intermediate tissues or poisoning of other parts of the body. Such an innovative high-precision access to the disease site is ensured with a likewise new technology of wires, catheters, and endovascular devices allowing safe percutaneous access, endovascular advancement, and specific treatment. A major advantage of this approach is a dramatically reduced burden of the treatment. In contrast to surgery, transcatheter therapies do not require deep anesthesia, invasive life support, prolonged wound care, and long-term rehabilitation whereas unlike pharmacotherapy they are not limited by multi-organ side effects and do not need to be repeated over and over again to achieve long-term benefits. Whereas it is yet to be seen whether these transcatheter therapies can successfully replace the conventional treatment options, they certainly are an attractive way to move forward where conventional surgery and pharmacology seem to have reached their limits or have very little potential to further improve treatment outcomes, e.g. in the control of elevated blood pressure (BP). Despite the phenomenal progress made over the past several decades in the development of antihypertensive drugs, only about half of the hypertensive cases can be effectively controlled to the target BP levels with this approach [1] mainly because of the development of drug resistance [2] and the inability of patients to strictly adhere to the complicated drug regimens [3]. The surgical attempts to treat hypertension were abandoned in the distant past because of excessive traumatism of the visceral surgeries [4, 5, 6]. Meanwhile, the progressive aging of the population causes a steady increase in the number of people with elevated BP that is expected to reach a total of 1.56 billion (1.54–1.58) in 2025 [7]. Thus, the already huge unmet need for hypertension treatment will only grow over time unless new and more effective therapies can reverse this deadly trend.

**Figure 1:**
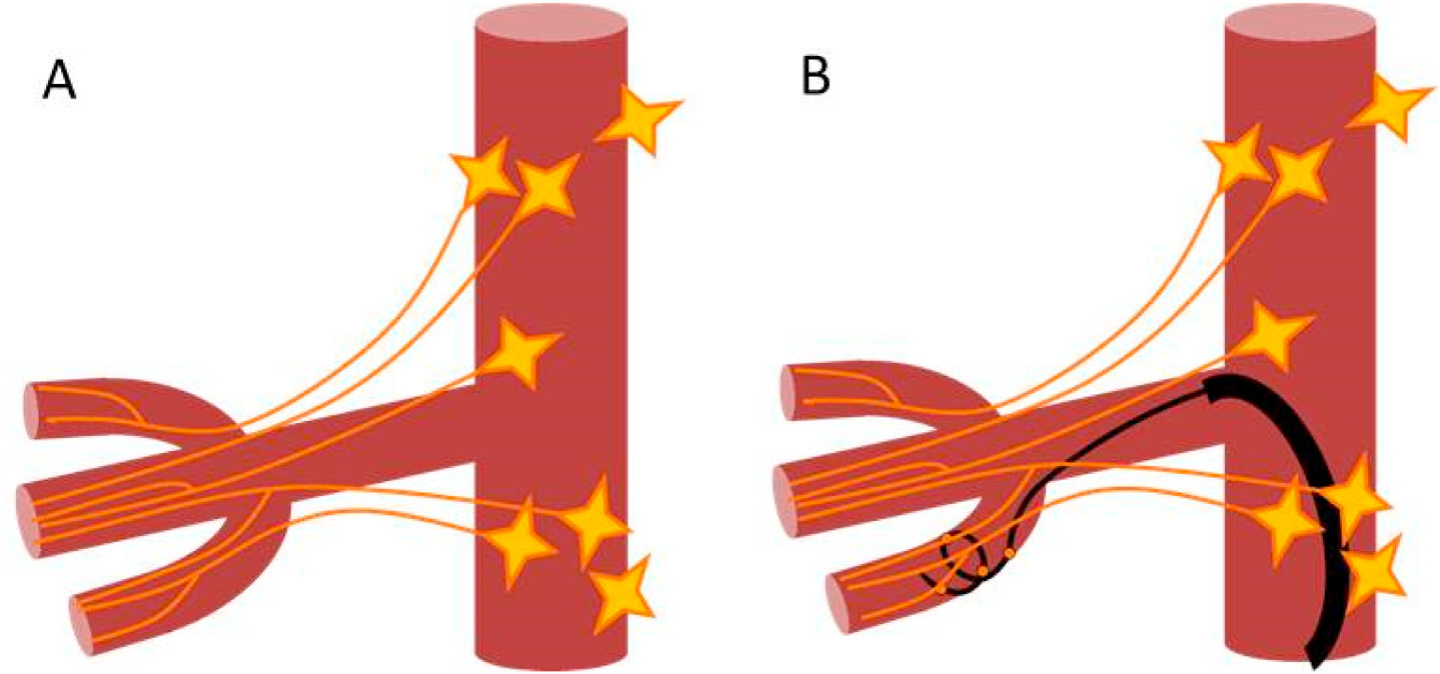
**A**. Actual anatomy of the renal nerve plexus. **B**. Anatomically optimized distal renal denervation.

Percutaneous renal denervation (RDN) is just such a step beyond conventional antihypertensive pharmacotherapy to innovative treatments of hypertension. Specifically, it is a remote transcatheter ablation of the renal nerves plexus performed using the specific catheter-based endovascular device, which is inserted percutaneously into the femoral or radial artery and advanced through the circulatory system into the renal artery where it delivers radiofrequency or ultrasound energy to the renal nerves surrounding the artery. The successful treatment creates a permanent conduction block through the renal nerves preventing the excessive sympathetic stimulation of the kidneys responsible for the maintenance of the increased circulating volume and pressure in hypertensive patients [8, 9, 10]. The well-studied physiology of the sympathetic system suggests a powerful BP-lowering effect of RDN in most clinical scenarios of hypertension. However, the first large sham-controlled clinical trial [11] of the therapy surprisingly failed to prove the antihypertensive efficacy of the early radiofrequency (RF) version of RDN. We linked this failure to the suboptimal anatomical mode of the treatment that was performed as 4–6 point applications of RF energy equally distributed in the main trunk of the renal artery assuming the likewise equal longitudinal and circumferential distribution of the renal nerves around the renal arteries. However, in reality, the renal plexus have been reported to have a triangular “fan-like” shape with a wide base near the aorta and apex converging at the kidney gate [12]. Thereby, the renal nerves are poorly accessible from the trunk of the renal artery but completely treatable from the distal branches. Following this anatomy, we developed a distal mode of RDN in segmental branches of the renal artery (Figure 1) and assessed the efficacy and safety of our distal RDN in comparison with the conventional main trunk RDN in a 6-month double-blind randomized controlled trial. The primary results of the trial were published in 2017 [13] and demonstrated the superiority of distal RDN over the main trunk procedure for the treatment of resistant hypertensive patients. Here we present the results of the extended (3 years) follow-up of our study that assessed the durability of the increase in BP-lowering effect of the therapy achieved by the anatomical optimization and, also, the long-term renal safety of the distal treatment performed very closely to the kidney.

## 2 Methods

A detailed description of the design and methods of the original study was published elsewhere [13]. In short, the study included patients of box sexes, aged 18–80 years, with apparent resistant hypertension (office systolic BP ≥ 160 or office diastolic BP ≥ 100 mmHg despite stable treatment with full doses of at least three antihypertensive drugs, including a diuretic). The patients were excluded if they had an established secondary cause of hypertension, 24h mean systolic BP < 135 mmHg, eGFR <30 mL/min/m [2], extended disease of the renal arteries, or severe comorbidity significantly increasing the risk of the intervention (investigator's assessment). All patients meeting the eligibility criteria were randomized 1:1 to either distal or conventional RDN in the Cath lab immediately before the procedure using a computer-generated randomization schedule. Treatment assignment remained unknown to patients, investigators, and other outcomes assessors for the entire study period. The study complies with all relevant ethical regulations for studies involving human subjects. Ethical approval was obtained from the Committee for Biomedical Ethics of the Tomsk Research Institute of Cardiology. All patients provided written informed consent prior to inclusion in the study. The study was registered in ClinicalTrials.gov (NCT02667912).

RDN was done using a Symplicity Flex radiofrequency ablation catheter and Symplicity G2™ generator through the femoral access. Conventional RDN was performed as a series of spot treatments at 5 mm intervals starting from the bifurcation and rotating the catheter by 45° after every single treatment. In the distal therapy group, the renal artery was deeply intubated and the ablation catheter was sequentially advanced into segmental branches of the artery where 2 - 4 separate point treatments were performed in each branch depending on its diameter (4 - if the branch had a diameter of 4 mm or greater and 2 - if the diameter was less than 4 mm). If less than 4 treatments were performed in the branch, 2 additional treatments were done in the distal trunk to achieve sufficient completeness of the intervention.

A total of 55 subjects were finally enrolled in the study and undergone RDN, 28 patients were treated by distal mode of the therapy, and the remaining 27 by conventional main trunk procedure. Baseline characteristics did not differ significantly between the groups except eGFR was slightly higher in the distal treatment group (Table 1).

**Table 1:** Baseline patients’ characteristics.

	Distal RDN	Conventional RDN
Number of patients	28	27
Age, years	56.5 ± 9.4	57.3 ± 9.5
Gender, males	10 (36%)	10 (37%)
Diabetes	14 (50%)	13 (48%)
CAD	12 (43%)	12 (44%)
Office sBP, mmHg	170·8 ± 23·0	169·4 ± 23·6
Office dBP, mmHg	93·4 ± 17·3	94·2 ± 19·6
24h mean sBP, mmHg	168·0 ± 24·1	158·0 ± 15·2
24h mean dBP, mmHg	91·6 ± 19·1	88·0 ± 17·6
Serum creatinine, μmol/l	79·3 ± 24·0	89·4 ± 20·7
eGFR, ml/min/m^2^	83·8 ± 23·4	70·6 ± 13·1∗
BP-lowering drugs, n	4·1 ± 1·1	4·3 ± 0·9
Diuretic use	96.4%	100%

∗ - p < 0.05, CAD – coronary artery disease, sBP – systolic blood pressure, dBP – diastolic blood pressure, eGFR – glomerular filtration rate (estimated by MDRD formula). Data are mean ± SD or (%).

The technical success of the procedure (at least 4 full-time applications of RF energy performed on each side) was 100% in both groups. The number of spot treatments delivered per patient did not differ between groups (13·6 ± 1·8 vs 12·7 ± 1·4 p > 0·05; distal versus main trunk therapy). Two subjects in the distal RDN group and 2 subjects in the group of main trunk treatment had accessory renal arteries that were successfully treated. No significant damage of the renal artery and its branches was found on intra-operational angiography. No major safety issues were observed.

During the blinded follow-up extension office and ambulatory blood pressures, changes in renal function (serum creatinine, eGFR) were assessed at 1 and 3 years post-procedure whereas the major renal adverse events (the new-onset kidney injury, that is persistent albuminuria/proteinuria and/or decreasing glomerular filtration rate (GFR) < 60 ml/min/1·73 m2, development of end-stage kidney disease with estimated GFR <15 ml/min/1·73 m2, and death from renal cause) were assessed over the entire three year study period. Variations in the concomitant antihypertensive drug therapy were assessed by the change in the average number of taken drugs. The blinding was maintained throughout the entire three-year study period.

The primary study outcomes were the change of 24-h ambulatory systolic BP from baseline to three years post-procedure (primary efficacy measure) and change in eGFR over the same period (primary safety measure). The secondary outcomes included changes of daytime, nighttime, office BPs, serum creatinine from baseline to one and three years post-procedure, and a number of the major renal adverse events over the entire study period.

Statistical Analysis: The significance of the differences in categorical data was assessed using the Chi-square test unless the expected values in any of the cells of a contingency table were <5 when the Chi-squared approximation was no longer adequate and Fisher's exact test was applied instead. For continuous data, we used Shapiro-Wilk's criterion to test the hypothesis of whether the data came from the normal distribution and applied t-tests (unpaired t-test or paired t-test) if this hypothesis was not rejected by the test. Otherwise, we applied the non-parametric Mann-Whitney U test for unpaired data and Wilcoxon signed-rank test for paired data. The 95% confidence intervals (CI) were calculated to assess the size of the treatment effects according to ICH E9 Guideline: Statistical Principles for Clinical Trials. Missing data were not imputed, and a per-protocol data set was used instead for the analysis of the follow-up data. A p-value <0·05 was considered significant, however, the final interpretation of p-values was in agreement with the ASA Statement [14]. The descriptive p-values were used to summarize the compatibility of the data with the study hypotheses rather than dichotomize the interpretation into “yes/no” categories.

## 3 Results

**Figure 2:**
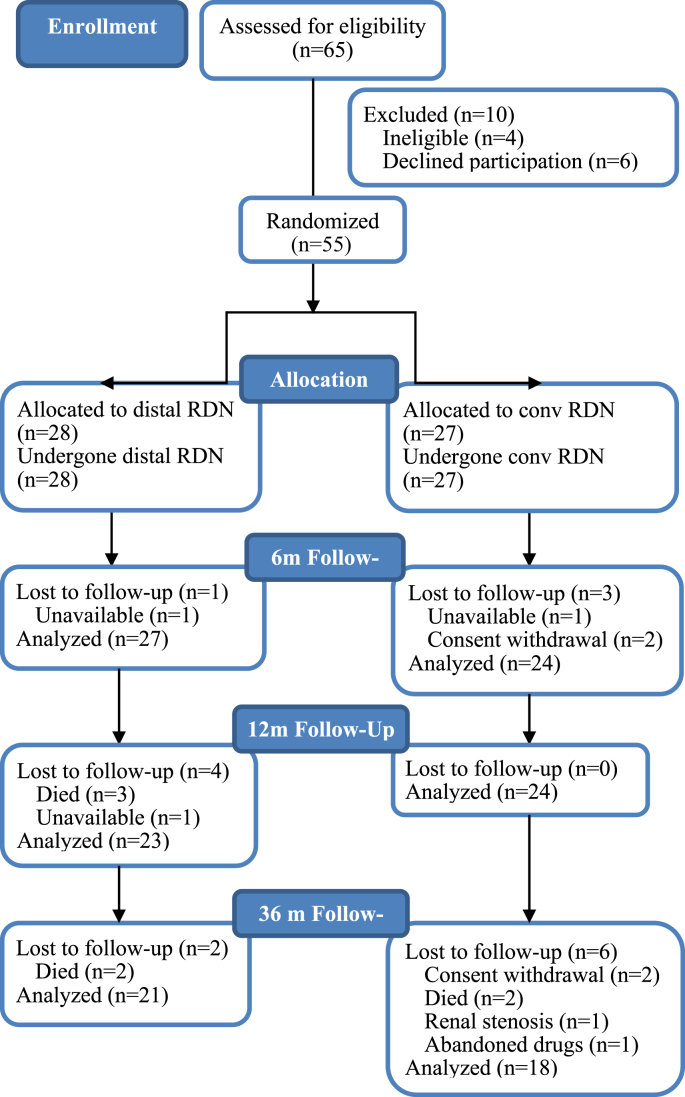
Study flow chart.

Forty-seven patients (85% of initial cohort) completed 12 months of follow-up, 23 after distal RDN, and 24 – after main trunk therapy. A three-year assessment was done in 39 patients (71%), 21 after distal RDN and 18 after RDN in the main trunk of the renal artery. The details of the patients' drop-out are summarized in the study flow diagram (Figure 2). In total, 16 subjects dropped out before the final three-year assessment. Seven subjects withdrew informed consent (two in the distal RDN group and five in the group of main trunk RDN). Seven patients died, five - in the distal RDN group (two cancer death, two fatal strokes, one death was caused by toxic shock due to severe enterocolitis that occurred more than 6 months post-procedure) and two - in the group of main trunk RDN (one fatal traumatic injury, one terminal heart failure). One subject in the group of main trunk RDN developed flow-limiting stenosis of the renal artery two years after the intervention; the lesion was successfully stented but the subject was excluded from the study. One subject from the same group totally abandoned the concomitant drug treatment before the one-year assessment and was also excluded from the analysis. Four non-fatal strokes occurred during the three-year follow-up, two - in the distal RDN group, two – in the group of main trunk treatment.

**Figure 3:**
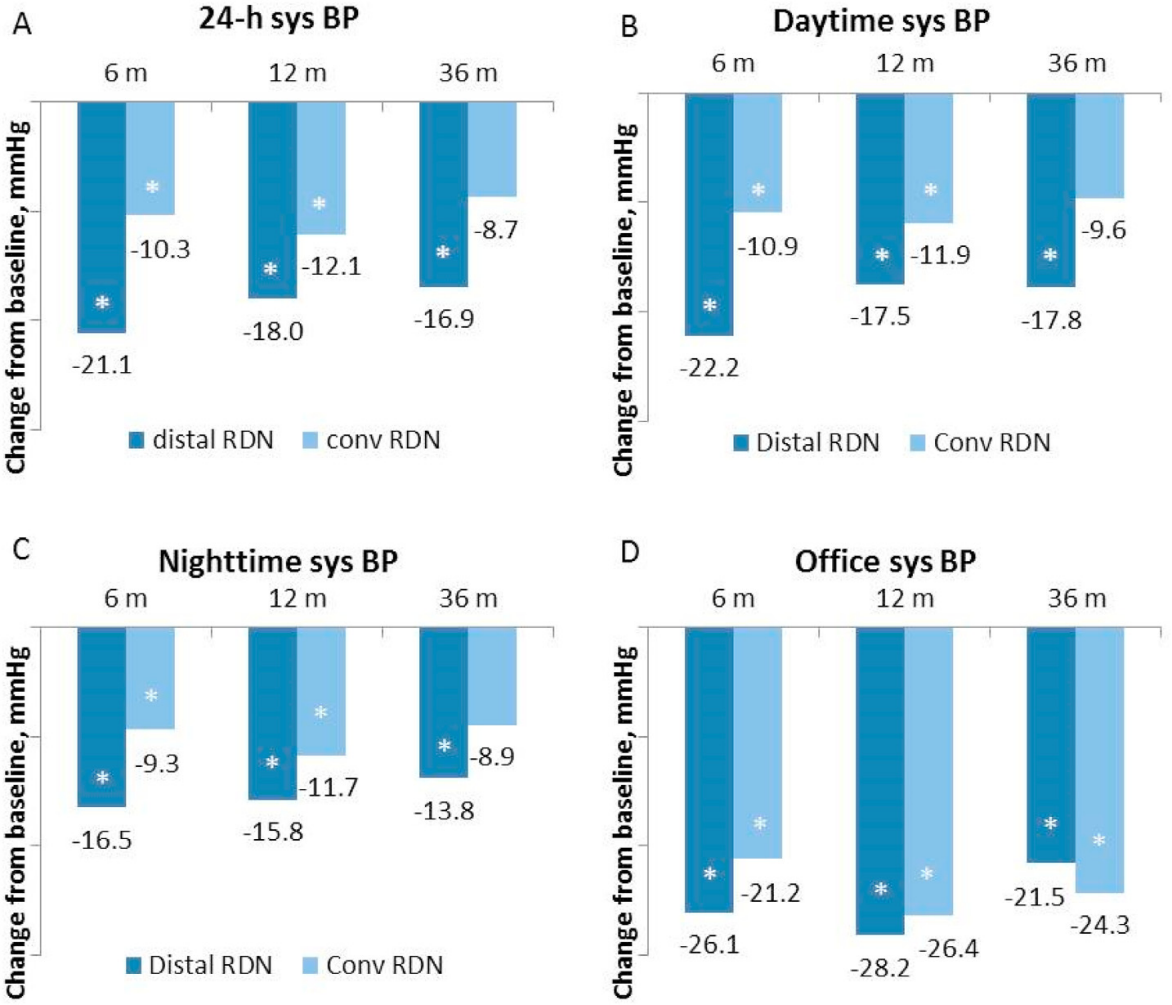
The decrease in office and ambulatory systolic BP at 6, 12, and 36 months after distal and conventional RDN compared to baseline. **A**. The decrease in twenty-four-hour mean systolic BP. **B**. The decrease in daytime mean systolic BP. **C**. The decrease in nighttime mean systolic BP. **D**. The decrease in office systolic BP. ∗ - p < 0.05; sys BP - systolic blood pressure.

The changes in blood pressure and renal function during the extended follow-up are summarized in Table 2. The between-group difference in changes of systolic BP throughout the study is also illustrated in Figure 3. In the group of distal treatment, the powerful initial drop of 24-h ambulatory systolic BP previously detected at 6 months post-procedure was maintained both at one-, and three-year assessments. All other office and ambulatory BP indices were likewise significantly lowered in this group during the entire three-year study period except the nighttime diastolic BP at the end of the study. At the one-year follow-up assessment, this powerful BP lowering was accompanied by a moderate drop in eGFR. However, from one to three-year eGFR slightly increased in this group despite the continuing strong BP lowering signaling some relative improvement in renal function during the late study period. In the group of main trunk intervention, the BP-lowering was quite moderate at one year with almost no change in eGFR. Subsequently, the BP-lowering effect further weakened and was only modest at three years post-procedure. In contrast, eGFR significantly decreased at three years after the conventional procedure compared to baseline (Table 2). The decrease of all ambulatory BP indices was remarkably greater after distal RDN than after the main trunk treatment both at one and three years post-procedure in contrast to office BP that decreased similarly in both groups.

**Table 2:** Change in BP and renal function compared to baseline.

		1 year			3 years		
		Mean	95%CI	p	Mean	95%CI	p
24-h sBP	distal	-18·0∗	[-27·6 to -8·5]	0.000	-16·9∗	[-27·3 to -6·5]	0.003
	trunk	-12·1∗	[-19·2 to -5·0]	0.002	-8·7	[-19·7 to 2·2]	0.058
24-h dBP	distal	-8·2∗	[-13·2 to -3·1]	0.001	-8·5∗	[-14·2 to -2·9]	0.005
	trunk	-8·1∗	[-12·8 to -3·4]	0.002	-5·8	[-11·8 to 0·2]	0.056
Day sBP	distal	-17·5∗	[-28·1 to -7·0]	0.001	-17·8∗	[-27·9 to -7·7]	0.001
	trunk	-11·9∗	[-20·0 to -3·7]	0.011	-9·6	[-20·9 to 1·7]	0.090
Day dBP	distal	-8·0∗	[-13·7 to -2·2]	0.003	-9·3∗	[-14·6 to -3·9]	0.002
	trunk	-8·0∗	[-13·4 to -2·6]	0.011	-7·2∗	[-13·9 to -0·5]	0.036
Night sBP	distal	-15·8∗	[-25·3 to -6·4]	0.001	-13·8∗	[-26·5 to -1·1]	0.034
	trunk	-11·7∗	[-19·6 to -3·8]	0.011	-8·9	[-22·0 to 4·1]	0.167
Night dBP	distal	-5·8∗	[-10·6 to -1·0]	0.007	-5·6	[-12·6 to 1·3]	0.106
	trunk	-7·7∗	[-12·8 to -2·6]	0.009	-4·8	[-12·0 to 2·5]	0.182
Office sBP	distal	-28·2∗	[-41·0 to 15·3]	0.000	-21·5∗	[-32·5 to 10·5]	0.000
	trunk	-26·4∗	[-36·9 to 16·0]	0.000	-24·3∗	[-37·8 to 10·8]	0.000
Office dBP	distal	-12·6∗	[-19·7 to 5·4]	0.001	-9·9∗	[-15·6 to 4·2]	0.002
	trunk	-9·7∗	[-17·9 to 1·6]	0.005	-13·2∗	[-20·2 to 6·1]	0.000
Creatinine, μMol/L	distal	9·9∗	[0·5 to 19·2]	0.043	6·3	[-1·8 to 14·4]	0.121
	trunk	2·0	[-6·3 to 10·4]	0.654	4·7	[-2·2 to 11·6]	0.211
eGFR, mL/min/sq.m.	distal	-8·9∗	[-14·8 to -3·1]	0.012	-6·5	[-13·2 to 0·3]	0.619
	trunk	-1·3	[-6·6 to 4·0]	0.714	-5·0∗	[-9·6 to -0·3]	0.037
Number of drugs	distal	-0·1	[-0·5 to 0·3]	0.358	0·4	[-0·3 to 1·0]	0.237
	trunk	0·2	[-0·3 to 0·7]	0.446	0·5	[-0·3 to 1·2]	0.177

∗ - p < 0·05 compared to baseline, sBP – systolic blood pressure, dBP – diastolic blood pressure, eGFR – glomerular filtration rate (estimated by MDRD formula). Data are mean and 95% confidence interval.

At baseline, the patients in the distal RDN group were taking slightly fewer antihypertensive drugs than those in the group of main trunk treatment. No significant changes were found in the average number of taken antihypertensive drugs in either group during the extended follow-up.

Eight patients developed new-onset chronic kidney disease during the three-year study period, five patients after main trunk RDN versus three patients after distal RDN. No other renal events were observed in either group.

## 4 Discussion

The assumption of equal longitudinal and circumferential distribution of the renal nerves around the renal arteries in humans is rather idealistic. On the contrary, several surgical reports published in the first half of the 20th century described the human renal nerve plexus as a triangular structure with a wide base near the aorta and apex converging at the kidney gate so that proximally the renal nerves pass at a significant distance from the renal artery but concentrate around its distal branches [12]. Thus, the equally distributed treatment in the main trunk of the artery may largely miss these nerves. We attempted to draw attention to the possible anatomical inadequacy of this mode of RDN early in 2011 [15] but had no data to support our belief. Therefore, we developed an anatomically optimized distal mode of the procedure by shifting the treatment from the main trunk to the segmental branches of the renal artery - the distal RDN. In addition to the relocation of the treatment, we modified the way of catheterization of the renal artery. Specifically, we used deep intubation of the renal artery with the guiding catheter advanced beyond the bifurcation, first, to stabilize the position of the electrode on the arterial wall limiting the electrode movement during pulse excursions, and, second, to selectively image the distal branches with reduced contrast volume (this is of great importance for RDN because the position of the catheter should be imaged at every treatment step, which significantly increases the volume of the contrast used per procedure compared to other interventions). Also, we developed a set of models of the optimal spatial distribution of the point treatments for major anatomical variants of distal branching of the renal artery (equal bifurcation, unequal bifurcation, trifurcation, etc.) and an algorithm to determine the optimal treatment configuration in case of atypical variants (e.g. short 1st grade branches, angulated branches, etc.). Then we tested our concept and method in 6 months double-blind randomized controlled trial that confirmed the superior efficacy of our distal RDN over the conventional main trunk procedure for treating resistant hypertension.

Parallel to our clinical trial, F. Mahfoud and co-authors experimentally confirmed that the treatment of the distal branches increases the efficacy of RDN [16]. However, in contrast to our trial, their experimental study included in addition to the similar groups of the main trunk or distal branches treatment, also, the third group of combined (main trunk plus distal branches) treatment, which produced the greatest effect. Based on these experimental findings the new randomized double-blind sham-controlled SPYRAL HTN-OFF MED trial implemented the combined treatment of the main trunk plus distal branches and was able to confirm in a rigorous sham-controlled fashion the significant BP-lowering effect of the RDN [17]. However, this approach has obvious disadvantages. The combined treatment of the trunk and distal branches significantly increases the number of spot treatments (from 11·2 ± 2·8 in Symplicity HTN-3 study to 43·8 ± 13·1 in SPYRAL HTN-OFF MED trial) and, proportionally, the duration of the procedure, X-ray exposure, contrast volume, dose of pain medications/sedatives, etc., which may override all the benefits of the increased efficacy of the treatment. Our study indicates that that the significant and durable improvement in the efficacy of the RDN may be achieved solely by shifting the anatomical location of the treatment without much increase in the number of the spot treatments. Another important finding is that distal treatment did not worsen the kidney function despite that both RF treatment and contrast injections were performed close to the kidney. Although the moderate decline in renal function occurred shortly after the distal RDN, the renal function was ultimately preserved in this group despite the continuing strong decrease in BP. In fact, there was an opposite trend to an increase of eGFR in the distal RDN group after one year. One possible explanation is that the strong drop in BP by about 20/10 mmHg after distal RDN naturally caused a proportional decrease in the renal perfusion pressure and glomerular filtration. Then, as the vasculature progressively adapted to the lower perfusion pressure by reverse vascular remodeling, vascular resistance gradually decreased and the renal perfusion was partially restored. The analysis of individual data from 2 large RCTs SPRINT and ACCORD-BP has shown that a decrease in BP due to antihypertensive pharmacotherapy causes a proportional decrease in eGFR, starting from a level of ≥10 mm Hg whereas lowering BP to less than 10 mm Hg does not affect eGFR [18]. Also, in participants with an initial >20% eGFR decrease, eGFR partially recovered after 12 months. Our study demonstrated similar effects of the RDN treatment: first, clear dependence of the change in eGFR on the magnitude of BP decrease; second, following an initial significant drop in the group of distal RDN eGFR recovered and the recovery likewise took place after 12 months. According to well-established renal physiology, the sympathetic denervation of the renal tubules, glomeruli, and vasculature should reduce the active sodium/water transport associated with high oxygen consumption and increase renal blood flow. The combined result of the reduced renal oxygen demand and increased blood and oxygen supply to the kidneys should have a nephroprotective effect in hypertensive patients vulnerable to kidney damage. The positive trend from one to three years after distal RDN and negative trend over the same period after main trunk treatment may indicate that the nephroprotective effect of sympathetic denervation requires sufficient completeness of renal nerve destruction, which is not achievable with the anatomically suboptimal main trunk procedure.

The study has important clinical implications. The obtained findings of the long-term efficacy and safety of the anatomically optimized RDN provide the necessary evidence for the adoption of this innovative therapy in wide clinical practice. In turn, adding new effective non-pharmacological treatment to the existing pharmacotherapy options has the potential to significantly improve blood pressure control especially in patients with poor tolerability of antihypertensive drugs, drug-resistant hypertension, or severe noncompliance, whereas the use of renal denervation as the first choice therapy in treatment naïve patients may significantly reduce the overall burden and cost of the antihypertensive treatment postponing the initiation of lifelong drug administration.

Several important limitations of the study should be taken into account. A small study sample limits the accuracy of estimating the size of BP-lowering effects, which may be different in a larger sample. Also, despite that at the end of the three year study period BP-lowering in the group of distal RDN still was twice as great as in the group of main trunk procedure the study was not able to confirm the long-term superiority of the distal RDN over the main trunk treatment due to several reasons including patient drop-out, reduction of absolute between-group difference in BP lowering and increase in variability of the individual BP changes. Another important issue is that concomitant pharmacotherapy could not be kept completely the same during 3 years, and even if the average number of taken drugs remained unchanged during the study, the effect of drug replacements and/or variable compliance could not be completely excluded. However, these variations are unlikely to explain the stable between-group difference in the BP-lowering over the entire three-year study period.

## 5 Conclusion

Thus, the study effectively confirms that anatomical optimization of percutaneous renal denervation by shifting treatment from the main trunk to distal branches of the renal artery results in a significant durable increase in the efficacy of the therapy without any compromise on its renal safety.

## Data sharing

Deidentified participant data will be available on request after publication. The data sharing will need the approval of the Committee for biomedical ethics and the Scientific council of the Research Institute of Cardiology of the Tomsk NRMC.

The original study protocol is available on the official website of the Cardiology Research Institute of the Tomsk National Medical Research Center at the following address https://www.cardio-tomsk.ru/storage/doc/gos.zadanie (in Russian).

## Declarations

### Author contribution statement

Stanislav Pekarskiy: Conceived and designed the experiments; Performed the experiments; Analyzed and interpreted the data; Wrote the paper.

Andrei Baev: Conceived and designed the experiments; Performed the experiments; Analyzed and interpreted the data.

Sergei Popov: Conceived and designed the experiments; Contributed reagents, materials, analysis tools or data.

Alla Falkovskaya, Ekaterina Sitkova, Irina Zubanova, Valeria Lichikaki, Musheg Manukyan, Mikhail Tarasov: Performed the experiments; Analyzed and interpreted the data.

Viktor Mordovin: Contributed reagents, materials, analysis tools or data.

### Funding statement

This research did not receive any specific grant from funding agencies in the public, commercial, or not-for-profit sectors.

### Data availability statement

Data will be made available on request.

### Declaration of interests statement

The authors declare no conflict of interest.

### Additional information

The clinical trial described in this paper was registered at ClinicalTrials.gov under the registration number NCT02667912.
